# Flexible Textile-Based Sweat Sensors for Wearable Applications

**DOI:** 10.3390/bios13010127

**Published:** 2023-01-12

**Authors:** Jing Yin, Jingcheng Li, Vundrala Sumedha Reddy, Dongxiao Ji, Seeram Ramakrishna, Lan Xu

**Affiliations:** 1 National Engineering Laboratory for Modern Silk, College of Textile and Clothing Engineering, Soochow University, Suzhou 215123, China; 2 Centre for Nanotechnology and Sustainability, Department of Mechanical Engineering, National University of Singapore, Singapore 117574, Singapore; 3 College of Textiles, Donghua University, Shanghai 201620, China

**Keywords:** textile, sweat sensors, sensor communication, wearable applications

## Abstract

The current physical health care system has gradually evolved into a form of virtual hospitals communicating with sensors, which can not only save time but can also diagnose a patient’s physical condition in real time. Textile-based wearable sensors have recently been identified as detection platforms with high potential. They are developed for the real-time noninvasive detection of human physiological information to comprehensively analyze the health status of the human body. Sweat comprises various chemical compositions, which can be used as biomarkers to reflect the relevant information of the human physiology, thus providing references for health conditions. Combined together, textile-based sweat sensors are more flexible and comfortable than other conventional sensors, making them easily integrated into the wearable field. In this short review, the research progress of textile-based flexible sweat sensors was reviewed. Three mechanisms commonly used for textile-based sweat sensors were firstly contrasted with an introduction to their materials and preparation processes. The components of textile-based sweat sensors, which mainly consist of a sweat transportation channel and collector, a signal-selection unit, sensing elements and sensor integration and communication technologies, were reviewed. The applications of textile-based sweat sensors with different mechanisms were also presented. Finally, the existing problems and challenges of sweat sensors were summarized, which may contribute to promote their further development.

## 1. Introduction

Traditional diagnosis based on physical hospitals shows limitations in obtaining and monitoring a patient’s physical condition in real-time. It is worth noting that the present physical health care system has gradually evolved into a virtual hospital form, which is not only time-efficient but also makes real-time diagnosis of a patient’s physical condition possible. With the increasing attention paid to health monitoring and the rapid development of electronic device technology, the customization of personalized wearable devices combined with personal conditions has attracted intensive interest. At present, most wearable devices are mainly used for monitoring changes in the physical conditions of the human body, such as heart rate, movement and temperature, etc., which hold limitations in reflecting the whole health status of the human body. Thus, the real-time monitoring of biological fluids such as bodily fluids, blood and interstitial fluid for physiological health evaluation has become a new trend in wearable sensors. Compared with the invasive analysis of blood components, monitoring bodily fluids (e.g., tears, saliva, sweat and interstitial fluid) as a non-invasive method is a more convenient, simple and safe way of obtaining physiological signals [1,2].

Sweat is the liquid secreted by the sweat glands of the human body and is distributed in all parts of the body [3]. It can be continuously collected and has great potential in body fluid monitoring by wearable sensors. Sweat production is influenced by a variety of factors, such as ambient temperature, physical movement, mental state, etc. Therefore, it is possible to use the related biochemical information in sweat to identify the physical and mental health of the human body [4]. The main components of sweat are water and NaCl in addition to some ions (e.g., K^+^ and Ca^2+^), metabolites (e.g., lactic acid, glucose and alcohol), etc. [3,5], and is dramatically dependent on the region of generation over the body [6,7]. Monitoring sweat components is one of the most important ways to adapt the physiological index in real time, which move from the bloodstream to the skin’s surface through sweat, carrying a lot of physiological information in the process [8]. For example, changes in the Na^+^ concentration in sweat can be used as a biomarker to monitor dehydration during long-term exercise [9,10], which can be an important implication for the water intake of athletes. In this way, a water and electrolyte deficit [6] caused by a significant loss of perspiration from heat sickness can be prevented [11]. Similarly, glucose changes in sweat provide a convenient way to detect blood glucose changes for diabetic patients, which is due to the correlation between the glucose concentration in the blood and the glucose concentration in the sweat [12]. Lactate buildup can cause soreness and fatigue in the body, and over a long period it can lead to severe illnesses such as body acidification or lactic acidosis [13]. Cortisol is considered to be one of the biomarkers used to monitor human mental health [14,15]. As a result, for these biomarkers, various types of wearable sweat sensors in different forms have been developed in recent years, such as headbands, wristbands, underwear and electronic tattoos with improved wearing comfort and flexibility. These sweat sensors are mainly based on electrochemical, conductive and colorimetric detection. Electrochemical detection mainly includes potentiometric, amperometric and voltametric detection and has the advantages of a high detection accuracy and a fast response speed [16,17]. Sensors based on conductivity changes in sweat can detect changes in sweat quantity and ion concentration, which can measure the conductance value directly or by using the electrochemical impedance spectroscopy (EIS) method. Compared with the above two methods, colorimetric detection is usually carried out based on visual observation and is easier to operate. Table 1 shows a comparison of the three different sensing mechanisms. On the other hand, the choice of materials has a great impact on the sensing mechanism of sweat sensors. For example, some natural materials are used for sweat delivery and enzyme fixation, while conductive materials are suitable for electrode preparation and detection based on electrochemical and conductive methods. The introduction of binding dyes can also enable colorimetric detection. In a word, their unique structure combined with specific materials can significantly improve the performance of sweat sensors.

In recent years, a large number of works related to various textile-based sensors have been reported, highlighting its rapid development. Wearable sweat sensors have been developed based on textiles owing to their advantages in flexibility, light weight and low cost compared to traditional conductor materials. Textiles are composed of natural or manufactured fibers, which possess the intrinsic characteristics of breathability, durability, sustainability and flexibility, meaning that their integration into sweat sensors generates little interference with daily life. As for their role in the preparation of sweat sensors, the unique structure of textiles can be directly used for sweat transportation channels. Textiles as sensing elements support the full contact of sweat with the fabric, which helps to increase the sensitivity of the sensor. Therefore, textile-based sweat sensors have great appeal and potential for real-time medical health and fitness monitoring.

**Figure 1:**
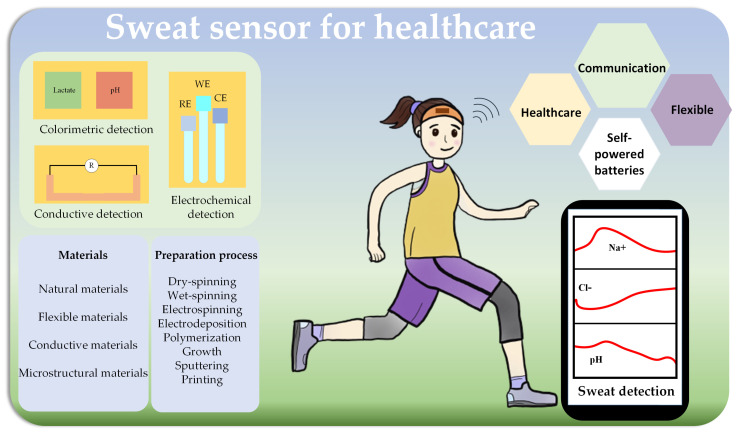
Illustration of textile-based sweat sensor.

Recent studies on textile-based sensors provide a reference angle for transforming laboratory-based testing into real-time wearable sensors. In this regard, we focused on the latest textile-based sweat sensors for wearable applications in this short review (Figure 1). Firstly, according to the material composition and component requirements of most sweat sensors, the materials and preparation processes of sweat sensors, transportation channels and collection, signal selection, sensing elements and integrating sensing with communication technologies were discussed. Next, the sensors used to detect different substances in sweat by diverse methods were briefly reviewed. Finally, we summarized the challenges and opportunities of sweat sensors to promote the further development of wearable sensors.

## 2. Materials and Preparation Processes of Textile-Based Sweat Sensors

Natural and functional materials processed into fibers, yarns or fabrics in various ways can be used to fabricate sweat sensors. Fibers composed of different materials can be combined and the desired properties of each material can be utilized to improve the overall performance of sweat sensors.

### 2.1. Materials

Natural materials including cellulose, silk, chitosan (CS), etc. have been explored as sweat sensors. These natural materials have the similar characteristics of being comfortable, breathable, flexible, unharmful and sustainable. Cellulose, as the most abundant renewable resource in nature, has been widely adopted in various forms for use in textile sensors with different structures such as fabric, yarn, etc., which can be used to collect or transport sweat [11,23]. For example, cotton fabric, as a direct sweat-transport material, keeps the skin comfortable under sweating conditions because of their good wicking rate [11]. Unlike cellulose, silk fibroin (SF) and CS can be used to modify electrodes for the immobilization of enzymes due to their unique properties. SF is a unique protein biopolymer composed of hydrophobic and hydrophilic segments. Studies have shown that SF can immobilize enzymes and maintain the stability of the enzymes [24,25,26]. The conversion of SF into a nanofiber structure results in a high specific surface area, which can immobilize the enzyme more efficiently and promote electron transport between the enzyme and the electrode, thus improving the sensitivity of the sensor [24]. Furthermore, carbonized silk fabric not only has a good electrical conductivity but also exhibits an enhanced electron transfer ability, which is especially practical for non-enzymatic sensor electrode materials [26,27]. CS is a kind of natural cationic polysaccharide biopolymer, which has good biocompatibility, biodegradability, hydrophilicity and antibacterial properties [28]. CS is often used as an immobilized substrate for enzymes, which not only helps to improve the ability of the enzyme against the action of metal ions [29] but also ensures facile electron transfer [30]. It has been reported that the combination of CS and graphene oxide (GO) as the electrode of a biosensor greatly improved the sensitivity of the sensor [29,31]. In addition to using natural materials for sweat transport, they can also be processed or combined with functional materials as electrodes.

Functional materials are roughly divided into flexible and conductive materials. Among them, the selection of flexible materials for the base substrate of wearable sensors demands that the material is more suitable for human skin and can meet the requirements of human daily activities. Flexible materials such as polyurethane (PU), poly(styrene-ethylene-butadiene-styrene) (SEBS), polyethylene terephthalate (PET), polyimide (PI), ecoflex, etc., are frequently used as substrates for sweat sensor, whereas conventional conductive materials, including conductive polymers (e.g., poly(3,4-ethylenedioxythiophene) (PEDOT), polyaniline (PANI), polypyrrole (PPy)), metal (e.g., Au, Ag, Cu), metallic oxides (ZnO), carbon, etc., have high conductivity, which is ideal for use as the electrodes of sensors.

Further developing these materials into microstructural forms such as quantum dots (QDs), nanoparticles (NPs), nanowires (NWs), nanorods (NRs) and nanotubes (NTs) as the electrodes of sweat sensors can effectively improve the sensitivity of the sensors. This is because these microstructures have a larger specific surface area than traditional conductive materials, which can effectively improve the sensitivity of the sensor as a sensing element. For example, carbon QDs (CQDs) are zero-dimensional carbon-based nanoparticles (<10 nm), which have a uniquely large specific surface area, strong electrochemical responses and a high electron transfer rate. In addition, they can effectively transfer electrons to electrodes. The immobilized content of an enzyme can be significantly increased by preparing the metal material into a microstructural form [32]. In addition, graphene (Gr)-based materials provide binding sites for enzymes when they are used as electrodes [31,33], which can effectively improve the sensitivity of the sensor [29], and they have great potential to be used in the field of biosensors. Among them, GO, due to the more oxygen-related functional groups on its surface, has been explored and developed for humidity-driven electric generator [34]. Some metallic microstructural materials such as Au, Ag, ZnO, etc., not only have a high electrical conductivity but also have antibacterial properties when integrated into wearable textiles. He et al. [35] reported that sweat sensors with Au nanodendrites as their electrodes have higher response signals than those with common bare Au electrodes. ZnO NRs coated on flexible carbon yarns as electrodes not only increase the surface area of the electrode, improve the hydrophilicity of the electrode and promote the rapid absorption of sweat but also bound the negatively charged enzyme on the electrode surface through the positively charged ZnO surface [36]. Integrating these materials into textiles by hybrid spinning, weaving, embroidery, printing or coating can effectively improve their flexibility and extend their application in flexible sensors.

### 2.2. Preparation Process

Textiles are used in sensors mainly in the form of fibers, yarns and fabrics. The combination of natural materials and functional materials processed into the form of textiles can effectively improve the performance of sensors.

#### 2.2.1. Direct Transformation

The preparation of functional materials directly transformed into fiber or yarns commonly adopts techniques such as dry spinning [37,38,39], wet spinning [40] and electrospinning (ES) [41,42], etc. The fibers or yarns are then incorporated into the fabric by weaving, embroidery or braiding [43]. With its simpler processing procedure compared to wet spinning, dry spinning prepares uniform and compact polymer fibers without using a coagulation bath [38]. Zhao et al. [39] successfully prepared SEBS/AuNWs fibers by dry spinning. In the preparation process, the SEBS/AuNWs were dissolved by a low-boiling-point solvent, and the AuNWs on the fiber surface effectively promoted the subsequent Au chemical deposition. For wet spinning, the polymer solution is injected into a non-solvent coagulation bath and solidifies to form continuous fibers. Zhou et al. [40] dispersed single-walled carbon NTs (SWCNTs) into sodium dodecyl sulfate (SDS)/poly(vinyl alcohol)(PVA) mixed solutions. Acetone was adopted as the coagulation solution in the wet spinning, which could remove the SDS surfactant. The SWCNTs were well aligned along the obtained filament axis, and the mechanical and electrical conductivity of the filament were also improved. Although these technologies have different preparation processes, they can be used to synthesize fibers by mixing different polymers.

Different from above technologies, micro/nanofibers have the advantages of a high specific surface area and porosity [42,44], which can improve the sensitivity of sensors based on these materials [24]. Furthermore, micro/nanofibers with core–sheath, beaded, spherical and porous structures can be prepared through the ES technique, which can be utilized in sensing [28], wound healing [44], filtering and so on. The nanostructure and high porosity attributed to electrospun micro/nanofibers could provide good conformity to the skin and facilitate sweat uptake through capillary action [1]. In addition to fabricating micro/nanofibers into membrane structures, studies have shown that converting nanofibers into yarn structures contributes to a higher sensitivity and more sweat diffusion channels [45]. 

Apart from prepared polymer fibers, pure metallic fibers or yarns are usually directly prepared by bundle-drawing or shaving processes [46,47]. The metallic yarns are then woven or embroidered into the fabric to extend its range of applications.

#### 2.2.2. Textiles Post Treatment

The diameter distribution of directly prepared fibers is sensitive to the aggregation and agglomeration of functional materials in the solution, resulting in nonuniform diameters and distributions of fibers loaded with functional materials. This is because excessive functional materials may destroy the properties of the substrate itself when mixed with the substrate. Therefore, materials coated on various types of substrates by electrodeposition [39,48], polymerization [49], growth [36], sputtering [1,36], dip-coating [50,51] and printing [52,53], etc., can effectively solve the above problems. Electrodeposition, polymerization and the growth of functional materials on substrate materials belong to the category of chemical processes. The electrodeposition technique is used to construct a variety of electrode materials, in which thin and uniform coatings are deposited on a substrate by redox reactions in a short time. The morphology of the deposited film can be adjusted by controlling the temperature and the applied current density as well as the electrolyte composition [54]. Peng et al. [12] deposited cotton-like Au microspheres on a carbon cloth by electrodeposition, which was more conducive to electrocatalytic applications. Yang and co-workers [55] electrodeposited PEDOT/GOx on the surface of Pt and Pt covered by PLLA nanofibers, respectively, and found that the nanofibers with Pt covered by PLLA effectively increased the GOx content and the sensitivity of the sensor and reduced the impedance of the electrode, which could be explained by the fact that the nanostructure increased the contact area, resulting in the positively charged PLLA and negatively charged GOx being better combined.

Another approach to coating functional materials on substrates is to use physical processes such as sputtering and printing technologies, which are simpler than chemical processes. Sputtering technology can achieve the deposition of metals or complex oxides onto a substrate material, and the cost is relatively low. In addition, the most commonly used coating method is the inkjet printing or screen printing of various materials on a substrate surface, which can achieve low-cost large-batch electrode preparation with a specific pattern. Although inkjet printing is less efficient than screen printing, electrodes with two or three different materials can be fabricated simultaneously by applying the same voltage pair with a certain spacing of ink drops, which greatly saves preparation time [53].

However, the materials and structures of textiles will affect the coverage of the conductive substances. Possanzini et al. [56] used cotton, silk and polyester threads as the substrates and coated PEDOT: PSS on the surface of the substrate material and compared its resistance value. Among the samples, the resistance value of cotton as the substrate was the lowest, and the resistance value of polyester as the substrate was the highest. Chung et al. [1] sputtered Au on a PU nanofiber and a PU microfiber surface and found that reducing the fiber diameter could ensure more cross hotspots per unit area and promote Au adhesion.

## 3. Key Components of the Sweat Sensors

The methods that sweat sensors use to analyze sweat mainly consist of sweat transportation channels and collectors, signal selection units and sensing elements. Some types of sweat sensors require the accumulation of collected sweat up to a certain volume for signal selection to detect specific or multiple substances.

### 3.1. Sweat Transportation Channels and Collectors

The effective transportation of sweat is supposed to not only deliver sweat to the sensing area but also provide a comfortable skin environment. Then, a sweat collection device can quantify the sweat loss and sweat rate that affects the concentration of ions. In recent years, various types of microfluidic devices have been prepared to collect sweat, but their preparation processes are complex. Wearable microfluidic devices utilize biocompatible, low-modulus elastomeric (e.g., PMMA, PDMS and its derivatives) substrates to prepare sweat capture patches for the pristine capture and clean storage of sweat [57,58,59]. These type of sweat collection devices are fabricated using films and only collect the sweat produced by sweat glands in specific locations, which is less comfortable than textile-based sweat collection devices [60].

**Figure 2:**
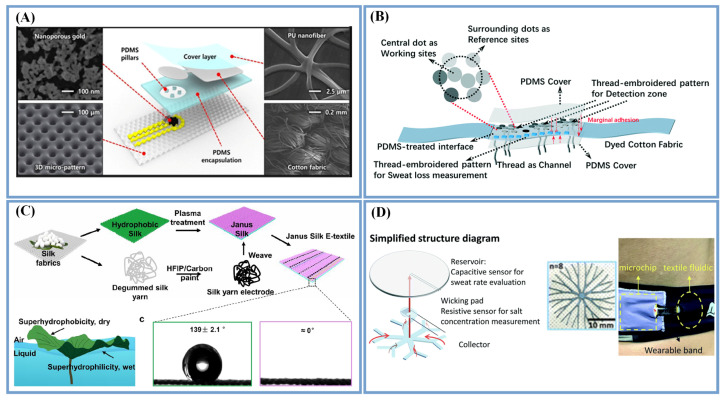
Sweat transportation and collection methods. Cotton fabric (**A**) (Reprinted with permission from Ref. [61]. Copyright 2019, copyright American Chemical Society) and threads (**B**) (Reprinted with permission from Ref. [62]. Copyright 2021, copyright Royal Society of Chemistry) directly used as capillary materials; (**C**) Janus textiles with the asymmetric structure of a lotus leaf (Reprinted with permission from Ref. [64]. Copyright 2021, copyright American Chemical Society); (**D**) Sweat collection device with three-layer structure based on plant roots (Adapted with permission from Ref. [11]. Copyright 2021, copyright Royal Society of Chemistry).

There are various strategies to fabricate sweat transportation channels and collectors based on textiles. For some textiles, the gaps between the fibers in the fabrics provide capillary channels where the liquid can be sucked along the thread [23]. For example, cotton fabrics or threads can be directly used as sweat channels [18,61,62] (Figure 2A,B). Bae et al. [61] directly used cotton fabrics as capillary materials, which had excellent liquid absorption and capillary flow abilities (Figure 2A). Cazalé et al. [63] prepared a transfer area and a storage area based on fibers for sweat sensors. The transfer area was made of non-woven polypropylene with a trilobal fiber structure, and the storage area was made of polyester/polyacrylate fiber, which could ensure a 300% water absorption by weight, while some other studies have also directly placed textiles near the sensing area for the real-time detection of sweat. However, the wicking behavior is bidirectional. The reason for this is that moisture can be delivered from the epidermis to the environment as well as in the opposite direction [64]. Therefore, coating the surface of the transport channel with a hydrophobic material opposite to the skin can prevent the sweat from evaporating. He et al. [65] coated PU on a cotton fabric band as a sweat transport channel, which could protect the liquid-wicking properties of the cotton fabric and avoid sweat evaporation. The average flow velocity reduced gradually from 0.32 cm s^−1^ to 0.13 cm s^−1^ as the band width changed from 4 to 8 mm. The material of the textiles has a great influence on their absorptive capacity and the evaporation rate of the liquid. For example, the absorptive capacity of textiles composed of natural fibers is greater than that of synthetic fibers [66]. 

Sweat transportation based on wetting gradients combined with the advantages of textiles and natural networks have been explored. Some Janus textiles, which imitate the asymmetric structure of a lotus leaf, have been used for sweat transportation, that is, a hydrophobic material is used on the side that touches the sweat to keep it dry, and a hydrophilic material is used on the other side to achieve the unidirectional transport of sweat to the collection device for substance detection (Figure 2C) [64]. Similarly, self-pumping Janus textiles created by the ES of a hydrophobic PU nanofiber array onto a superhydrophilic microfiber network can unidirectionally and fully transport sweat from the skin to the external electrode side [35]. Silica NP–TPU composite nanofibers have been introduced as sheaths for improving the hydrophilicity over the superhydrophobic SEBS substrate for the in situ concentrating and collection of sweat [67].

Furthermore, sweat transportation and collection inspired by the water transport principle of plant roots has also been reported. For instance, Chen et al. [11] designed a sweat collection device with a three-layer structure. As indicated in Figure 2D, the bottom layer adopted a fast wicking - textile material with a fractal structure, the middle layer had a flow channel as the detection area and connection and the top layer had a disk-shaped fabric reservoir. The effective combination of the three layers can be used to detect the change in sweat volume, rate and concentration.

### 3.2. Signal Selection Unit

Signal selectivity, as an important factor that affects the accuracy of a sensor, is the ability to resist the interference and disturbance from other substances in the detection environment. One way to eliminate the unwanted disturbance is to select the target substances that need to be detected and exclude the interference of other ions. Polyvinyl chloride (PVC) and PU have been reported to be ion-selective membranes (ISMs) [52,68] or ion-sensitive field effect transistors (ISFET). The corresponding ionophore, ion exchanger, polymer matrix and plasticizer are dissolved in a specific solvent and are then dried to form an ISM [69]. The ISM is deposited or coated on conductive yarns or fabrics as the electrodes, which can achieve a signal response to target ions without interference from other ions [70]. Textiles as electrodes have a hierarchical and porous structure, which facilitates the rapid diffusion of electrolytes and the efficient transport of ions and electrons [71]. Another way is that certain components or structures can act to block interfering substances. The limitation of ion contamination can be eliminated by employing alkaline conditions on the electrode surface, as the OH¯ ions suppress the adsorption of Cl¯ ions to the electrode’s surface [72]. Nanoporous polyamide (PA) membranes used as a substrate material can act as a biomolecular sieve, allowing only desirable biomolecules to travel to the sensor surface, thereby preventing interference from other factors and improving the accuracy of the sensor [73].

In addition to the selection of specific substances, some materials such as CNTs [74] and PEDOT: polystyrene sulfonate (PEDOT: PSS) [75,76] can convert the charge carriers from ions to electrons, namely, ion-to-electron transducers. For example, PEDOT: PSS can convert the charge carriers from ions (e.g., Na^+^, K^+^) to electrons via the doping/de-doping of PEDOT: PSS.

### 3.3. Sensing Element

The selection of the material and structural design of sensing elements for sweat sensors are vital, since they have direct impacts on the sensor’s sensitivity and electrical performance. The electrodes in sweat sensors, as crucial elements, especially for sweat sensors based on electrochemical methods, are mainly composed of either two-electrode systems (a working electrode (WE) and a reference electrode (RE)) or three-electrode systems (WE, RE and counter electrodes (CE)). Most sweat sensors respond to metabolites in sweat, and the principle involves the anchoring of enzymes to the WE. Therefore, the selection of the materials and structural design of the electrode has a great impact on the sensitivity and selectivity of the sensor. Traditional sweat sensors consist of screen-printed electrodes on rigid substrates such as glass or plastic. The rigid substrates restrict the flow of the capillary tube and have poor flexibility, which requires further consideration for their integration into clothing [43]. Incorporating conductive materials into fibers with unique structures as electrodes is beneficial for improving their flexibility. Li et al. [48] deposited different active materials onto CNTs, forming a coaxial structure, which could test physiological signals. Transforming Ag/AgCl into fibers as electrodes has more stable potential than commercial solid-state Ag/AgCl electrodes.

Natural materials are not conductive by themselves, but they can be used as electrodes by simple treatment or the doping of conductive substances. For example, He et al. [71] transformed silk fabrics into highly conductive, intrinsically nitrogen-doped, flexible and structurally maintained carbon textiles with a graphitic nanocarbon structure through thermal treatment. The treated silk fabric was used as an electrode with abundant active sites, facilitating electron transfer, the quick diffusion of electrolytes and efficient ion and electron transmission. Growing or coating conductive materials on natural threads or yarns is also an effective approach for integrating them into textiles. Smith et al. [77] prepared highly conductive cotton fibers as an electrode by dipping them into PEDOT: PSS ink, multi-walled CNTs (MWCNTs) and PEDOT: MWCNTs. The conductive cotton prepared by different dispersion methods had a great impact on its electrical conductivity, mechanical properties and the subsequent deposition of PANI. The PEDOT-MWCNT-coated cotton fibers showed an impressive flexibility and a high conductivity.

The structure of electrodes or the sensing area of the sensors is important for sensor performance as well. For instance, the porous structure of mesoporous carbon results in a high electron transfer rate, which interacts with the GOx enzyme with an increased surface area and enhances the sensitivity to glucose [72]. AuNP-modified electrodes can improve the charge transfer rate and glucose sensitivity compared to common Au electrodes [78]. Designed electrodes based on textile structures have larger contact areas than traditional electrodes, which effectively improves the sensitivity of these electrodes. Wang et al. [68] fabricated ecoflex/polytetrafluoroethylene (ecoflex/PTFE) fibers with a unilateral bead structure and then coated CNT film and AuNPs on the ecoflex/PTFE fibers as electrodes in sequence. The fabricated ion sensor had both a high stretchability and a high sensing stability due to the low strain within the bead region of fiber.

To solve the damage to electrodes caused by scratches or mechanical breaks during exercise, some interesting works have proposed the preparation of self-healing sensor electrodes [76,79]. Yoon et al. [76] prepared a carbon fiber thread coating with a citric-acid-based supramolecular polymer, which exhibited highly sensitive and self-healable properties and could rapidly self-heal within 30 s at room temperature.

### 3.4. Integrating Sensing with Communication Technologies

In recent years, many related sweat biomarker detection methods have been proposed. In order to identify the multiple biomarkers in sweat using wearable sensors in real time and in a portable manner, it is necessary to integrate sensing technology with communication and analysis technology on flexible printed circuit boards (FPCB) to realize the transmission of signals to external receiving devices for users to obtain the information. The most commonly used communication technology includes near-field communication (NFC) [80] and Bluetooth modules [69], although they are only suitable for close-range signal transmission. The advantage of NFC is that it does not rely on battery power to transmit signals wirelessly, but it can only be used at very short distances [80]. Bluetooth communication usually demands a high power consumption and needs to be combined with low-power instruments for proper operations [16]. These communication technologies can realize the internet-of-things approach of transferring data to the cloud [81].

Therefore, batteries play a key role in the stable operation of sensor in terms of communication. Traditional batteries, such as Li-ion batteries, coin cells, etc., have been rigidly compared with flexible batteries and are difficult to integrate into miniaturized sensors, which limits their application in wearable sensors. By combining them with self-powered batteries such as triboelectric nanogenerators (TENGs) [82], piezoelectric generator [83], biofuel cells (BFCs) [84,85,86], etc., the monitoring of sweat information can be achieved without an external power supply. 

**Figure 3:**
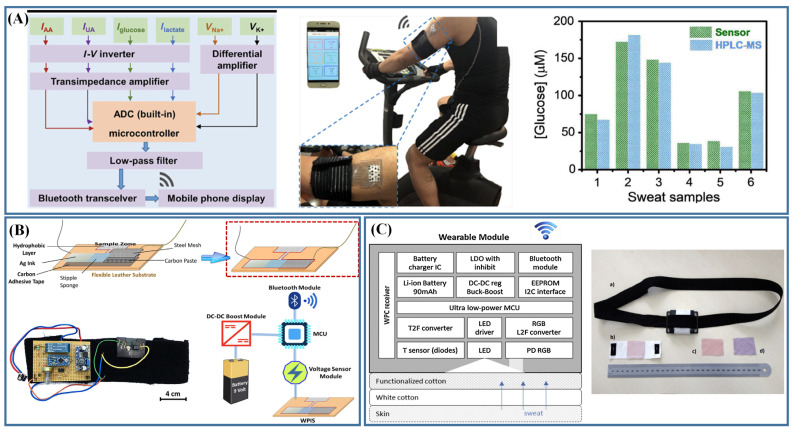
Sweat sensors integrated with communication technologies. (**A**) Sweat sensor based on electrochemical detection was applied for real-time multiple analysis of sweat [71]; (**B**) Sweat sensors based on potentiometric detection and signal readout circuits were embedded into a wearable sweat headband (Reprinted with permission from Ref. [87]. Copyright 2022, copyright American Chemical Society); (**C**) The main components of wearable system based on colorimetric detection (Reprinted with permission from Ref. [88]. Copyright 2016, copyright Elsevier).

Sweat sensors combined with communication techniques have great potential to break through traditional lab-based analysis methods and achieve stable and fast detection in dynamic situations. Sweat sensors based on electrochemical detection with multiplex sensing arrays integrated with signal transducers, conditioning, processing and Bluetooth modules for sweat analysis that transmit information from the sensors to a smartphone [71]. The fabricated sweat sensors can simultaneously track six biomarkers in the sweat of the user (while in motion) in real time (Figure 3A). Ghoorchian et al. [87] prepared a sweat sensor based on potentiometric detection, which was combined with a readout circuit to transmit the signals to a tablet in real time (Figure 3B). The readout circuit contained an Arduino Pro Mini, an Arduino Voltage Sensor Module, a DC Boost converter, a Bluetooth module and a 9 V battery. The sensor measured the conductivity changes in the sweat by the EIS method. It has also been reported that a digital-to-analog converter was used to generate a sine wave to the sensor, and the impedance was calculated by measuring the amplitude and phase shift of the wave at the other end of the sensor. Then, the measured data was converted into a digital code [11]. Liu et al. [59] combined an oscillator, a sweat sensor based on EIS measurement and a microcontroller with a Bluetooth transceiver on PCB to form a wearable sensor, which could transmit data to a phone and then convert these data into conductivity values.

Sweat sensor based on colorimetric detection can also achieve real-time and continuous detection. In addition to the colorimetric use of the naked eye, RGB values can be directly obtained, or images can be directly transmitted for color matching by communication technology [20,88]. Caldara et al. [88] integrated color sensors with embedded processing devices and Bluetooth radios, which could realize measurement control and continuous data uploading. As shown in Figure 3C, the color sensor integrated a photodiode with the L_2_F principle to collect the maximum reflected light from the fabric to a readout RGB value. The system was capable of detecting any color in the visible spectrum and could identify different colorimetric dyes. At the same time, combined with ultralow-power-consumption microcontroller, the sensor could effectively improve the working time. 

## 4. Applications in Wearable Sensors

The detection and sensing of abundant substances in sweat as well as the generation of energy are considered to be very promising green wearable sensor systems. Table 2 lists the common components of sweat and their associated diagnosis applications. To detect the target substances in sweat, the commonly used methods are the electrochemical, conductive and colorimetric methods, as indicated in Table 3. In addition to testing the composition of sweat to analyze human health, recent works have proposed the use of biofuels in sweat as BFCs or to develop self-powered sensors based on sweat.

### 4.1. Sweat-Quantity-Sensing Devices

Sweating is one way in which the body temperature is regulated [19]. The real-time monitoring of sweat volume or the sweat rate helps to evaluate the dehydration or abnormal sweating of athletes in high-intensity exercise and workers in extreme conditions. To achieve this, the sweat volume or rate can be directly measured by sweat transportation or collection. Li et al. [60] conducted plasma treatment on cotton thread to effectively improve its hydrophilicity and wicking ability, achieving a sweat delivery rate of 0.27 ± 0.03 cm/s. In addition, the effective combination of hydrophilic and hydrophobic materials to form a certain pattern can evaluate the sweat volume or rate. Zhao et al. [62] adopted hydrophilic cotton thread embroidered on a substrate of hydrophobic cotton fabric in the form of color blocks as microchannels and micro-reservoirs for sweat collection and detection. A total of 12.5 μL of sweat could be collected for each color block. Chen et al. [11] prepared a sweat collector with a capacitive sensor to determine the volume of sweat based on the capacitance value.

**Figure 4:**
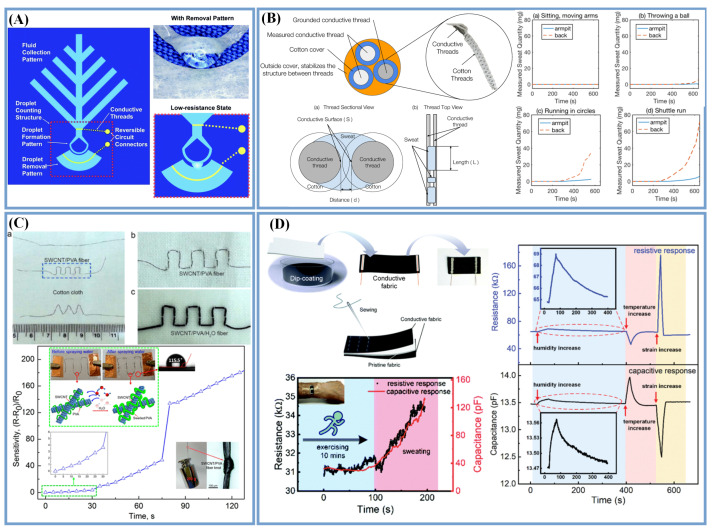
Various sweat-quantity-measuring and sensing devices. (**A**) The sweat forms a connected/low resistance state between the two fabrics (Adapted with permission from Ref. [98]. Copyright 2017, copyright Royal Society of Chemistry); (**B**) The conductivity between the three conductive threads varies according to the amount of sweat absorbed [19]; (**C**) Resistance changes by expanding PVA molecular chains by absorbing water molecules (Adapted with permission from Ref. [40]. Copyright 2017, copyright American Chemical Society); (**D**) Humidity sensors integrated with resistance and capacitance (Adapted with permission from Ref. [51]. Copyright 2021, copyright Royal Society of Chemistry).

Sweat contains ions that make sweat conductive, and this can be used to measure an individual’s perspiration quantity. Yang et al. [98] laser-processed a hydrophilic pattern on a superhydrophobic fabric, where the through-hole structure on the fabric allowed the fluid to pass through the fabric and be transported to the hydrophilic pattern on the surface of the fabric (Figure 4A). As the volume of sweat increased, when the droplets reached the removal pattern, the two fabrics were connected by the sweat to form a connected/low-resistance state, so that the sweat flow rate could be monitored in real time according to the change in resistance. Cotton thread was used as the conductive thread, which could change the electrical conductivity of the woven cotton fabric by absorbing sweat. Therefore, the quantity of sweating can be evaluated based on the electrical conductivity changes of the cotton thread. Jia et al. [19] proposed a sweat sensor based on three conductive threads wrapped in cotton, which were woven into a stable triangular structure to detect the quantity of sweat. The conductivity between the conductive threads varied according to the amount of sweat absorbed. As shown in Figure 4B, sensors were used to measure the sweating in the armpit and on the back during different movement states. It was found that the sweat produced in the same part had a similar variation trend under different exercise states, and the amount of sweat on the back was more than that in the armpit.

There have been several reports of prepared humidity sensors that can be used to evaluate the quantity or flow rate of sweat [40,99,100]. Salvo and co-workers first integrated sweat sensors on a textile substrate using two commercial humidity sensors placed on the fabric at different heights from the skin surface, which could evaluate the sweat rate of the human body by measuring the flow of water vapor [101]. In order to make a sensor completely based on textiles, some researchers fabricated a humidity sensor consisting of two parts: a moisture-sensitive material and a conductive material, and they estimated the amount of sweat by detecting the change in the resistance of the composite material. Zhou et al. [40] fabricated an SWCNT/PVA filament with a high hydrophobicity by the wet-spinning technique. As shown in Figure 4C, the conductive SWCNT network was adjusted by absorbing water molecules to expand the PVA molecular chains, such that it exhibited resistance changes at different relative humidity in a short response time of 40 s. Yang et al. [51] presented a textile-based sensor with an integrated resistance and capacitance by a simple dip-coating and sewing method. As shown in Figure 4D, carbon particles were coated on nylon/spandex fabrics as the strain-sensing layers, endowing the elastic fabric with abilities for sensing strain, temperature and humidity changes. As the sweat was absorbed into the fabric, the distance of the carbon particles increased, resulting in a change in the fabric’s electrical resistance. To distinguish multiple signal stimuli, a sandwich-formed capacitive sensor was fabricated by sewing. The addition of sweat increased the permittivity of the capacitive sensor, thus causing the capacitance value to increase. The fabricated sensor had the same tendency to sense the humidity in the sweat, and as the humidity increased, both the capacitance and resistance had a positive response.

### 4.2. Ion-Sensing Devices

Sweat contains a large number of micronutrients such as Na^+^, Cl^−^, K^+^, Ca^2+^, etc., which endow sweat with natural, safe and reliable electrolyte properties. These ions have a tight connection to the heart rate, blood pressure, cardiovascular function, muscle contraction, enzyme activation and bone development in humans [45,50]. The concentration of Na^+^ is the highest among all the ions, with a typical value of 38 mmol/L [88]. Therefore, changes in the Na^+^ concentration in sweat can enable the monitoring of heat stress and the detection of various diseases such as hyponatremia and cystic fibrosis, which can provide important information for clinical diagnosis [87]. 

The most-used detection method for detecting the change in ion concentration in sweat is the electrochemical method, which uses ion-selective electrodes (ISE) to detect the target ions. Kil et al. [102] printed serpentine-patterned Gr/ecoflex electrodes onto socks, which showed a high conductivity and stable resistance under a strain of 150%. A Na^+^-ISM was drop-cast onto this Gr/ecoflex electrode, which could exhibit an excellent response to the Na^+^ concentration under different motion states. Similar toISMs, binding ion-specific receptors is a way to detect target ions. Ghoorchian et al. [87] used Na_0.44_MnO_2_ as the sensing material to monitor Na^+^ in sweat, which enabled the observation of a stable potentiometric response for Na^+^. Combined with a microcontroller-based circuit integrated into a headband, a wireless transceiver could be used to real-time monitor the Na^+^ during exercise activity. Ag/AgCl can also be used as an electrode to measure sweat Cl^–^ levels directly [56,103]. Possanzini et al. [56] coated PEDOT: PSS onto four different types of threads to enable the threads to exhibit a semiconductor behavior and then deposited Ag/AgCl onto these threads with semiconductor behaviors as specific receptors, which could respond the current of the threads with the change in the Cl^−^ concentration without performing output signal processing or correction. In addition, AgCl exhibits a reproducible and well-defined electrochemical potential with respect to a RE, which depends on the concentration of Cl^−^. Mo et al. [45] prepared core–sheath yarns using nylon yarns as the core layer and PAN/PVP/valinomycin nanofibers as the sheath (PPVN yarns). The hydrophilic PPVN yarns were woven with hydrophobic polyester yarns to form a plain fabric so that the sweat was completely confined in the PPVN yarns, which helped to obtain a stable and accurate signal. Among them, the PPVN yarns as a specific receptor for K^+^, the valinomycin was highly selective for K^+^ and generated the corresponding potential.

Different from the above method, cotton fabrics were dipped into a mixture solution of reduced GO (rGO) and aminofullerene (C_60_) spheres to prepare sweat sensors with specific ion-sensing functions. The resistivity of the sensors increased with the increase in the cation’s (Na^+^, K^+^, NH_4_^+^, Gd_m_^+^) or anion’s (Cl^−^, I^−^, NO^3−^, ClO_4_^−^) radius [50]. 

Saline concentration can be estimated from impedance measurements without ISEs. The impedance is sensitive to the saline concentration [11], which can be used as one of the means to detect sweat. This technique requires placing an interdigital electrode in a sensing area to detect the salt concentration based on the impedance with a frequency of 10 Hz, which shows a fast response time to abrupt changes in sweat rates or concentrations.

### 4.3. pH-Value-Sensing Devices

The pH value of sweat in a healthy human usually varies from 4 to 7, which has become one of the biomarkers for monitoring exercise intensity [1]. It has been reported that there is a relationship that exists between the pH value and the concentration of Na^+^ [104]. The higher the pH, the greater the concentration of Na^+^. A colorimetric approach is always used for sweat pH measurement. Screening this color change by the naked eye or via a spectrometer can enable the monitoring of sweat status in real-time during exercise. It is often valuable to use color-changing materials blended into a textile to monitor the acidity of sweat. This method involves a pH-sensitive dye and halochromic materials, which change color depending on the pH of the sweat. These materials are polyelectrolytes possessing acidic or basic groups in their structure, which will either accept or release protons depending on the environmental pH [105,106]. 

**Figure 5:**
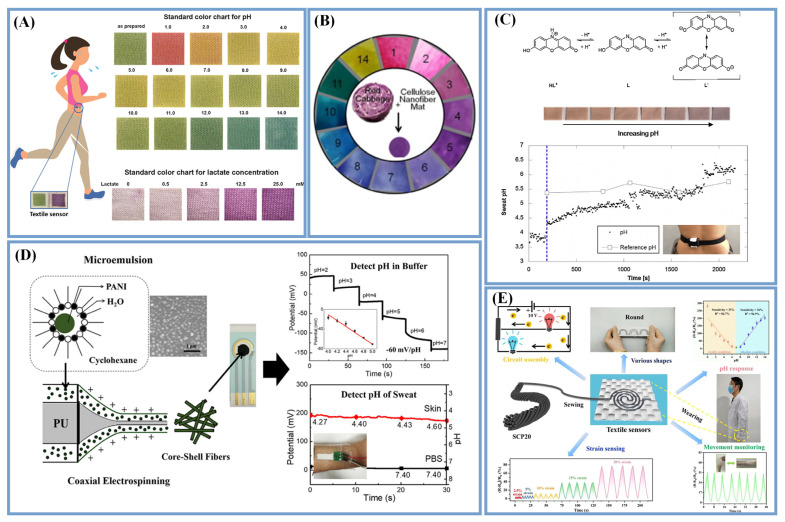
pH sensors based on different detection methods. (**A**) pH values between 0 and 14 could be distinguished by the color changes (Reprinted with permission from Ref. [22]. Copyright 2019, copyright Elsevier); (**B**) Anthocyanins as active dyes immobilized on the electrospun cellulose acetate nanofibers (Reprinted with permission from Ref. [41]. Copyright 2015, copyright Elsevier); (**C**) Environmentally friendly halochromic dye (Adapted with permission from Ref. [88]. Copyright 2016, copyright Elsevier); (**D**) PANI and PU core–sheath fibers (Adapted with permission from Ref. [111]. Copyright 2020, copyright Springer Nature); (**E**) The resistance of composite yarns response to pH value (Reprinted with permission from Ref. [113]. Copyright 2022, copyright Elsevier).

In addition to conventional pH indicators, pH-sensitive dyes have been reported recently, including bromocresol purple (BCP), nitrazine yellow (NY), anthocyanins [41,107], tricyanofuran hydrazones [108], a mixture of bromocresol green (BCG) and methyl orange (MO) [22] and so on. Most synthetic dyes are halochromic dyes, which have adverse effects on the environment and are also allergenic to humans [106]. Adding hydrophilic materials such as CS, cellulose, GO or porous structures of materials can boost their sensitivity and shorten their response time dramatically. Prophet et al. [22] deposited CS, sodium carboxymethyl cellulose (NaCMC) and dye (a mixture of MO and BCG) on cotton fabric in sequence and added CTAB to fix the dye to prevent fading caused by the interaction between the positively charged amino group of CS and the negatively charged indicator dye. The L* a* b* space model was used to quantify the color change, where the value of L* represented the brightness, while the values of a* and b* represented the distance along the red–green and blue–yellow axis, respectively. As shown in Figure 5A, the pH values between 0 and 14 could be distinguished by the different colors. Schueren et al. [109] blended NY into a polycaprolactone/CS solution to fabricate electrospun pH-sensitive biocompatible nanofibers. Devarayan et al. [41] immobilized anthocyanins onto electrospun cellulose acetate nanofibers, causing significantly difference in colors at all PH values (Figure 5B). Furthermore, the prepared sensors could be stable over long periods at different temperatures, and the color was reversible, meaning that the sensors could be recyclable.

Halochromic textiles have been successfully used in pH sensors and have the characteristics of durability and reversibility. Li et al. [88] adopted an environmentally friendly halochromic dye (litmus) with 3-glycidoxypropyltrimethoxysilanein to form hybrid sol–gel matrices to impregnate cotton fabrics that affected their protonation equilibria. As shown in Figure 5C, the 7-hydroxyphenoxazone in litmus could combine with a proton to yield the red cation HL^+^ in an acidic solution, while in basic solutions, it eliminated a proton to give the blue L^-^ species. The pH of the sol–gel-based wearable sensor fitted reasonably well with the commercial pH probe reference measurements. Yapor et al. [110] prepared nanofibers by incorporating 10,12-pentacosadiynoic acid (PCDA) into PU, and then PU–PCDA nanofiber composites were obtained via photopolymerization under UV irradiation,, which demonstrated excellent colorimetric responses to the presence of *E. coli* and changes in pH as external stimuli.

Some materials with electroactivity such as PANI, IrO_2_, etc., have a high sensitivity to PH in terms of their electrochemistry. PANI is often used to prepare pH sensors [48,77,111,112] and shows larger zeta potential changes at different pH values due to the protonation changes on the PANI surface. Hou et al. [111] developed PANI and PU core–sheath fibers through coaxial ES. Using PU as the core layer could improve the mechanical properties of the sensors, and the mechanical properties and the conductivity of the sensor could be changed by adjusting the proportion of PANI and PU. As shown in Figure 5D, the PANI/PU sensor responded to different potentials at different PH values and was stable in terms of deformation, temperature and detection time and could thus be applied for sweat PH detection. Hou et al. [79] developed a self-healing and flexible PH sensor by coating self-healing polymers (SHP) on the surface of PANI/carbon fiber threads. The fabricated sensor had a high sensitivity and a self-healing efficiency of 97.8% within 30 s, which ensured the stability and reliability of the sensor during operation. The measured results obtained were equivalent to those generated by commercial PH meters. The functionalization of rGO fibers with ferrocene showed clear redox peaks, which displayed a linear range of pH 3–8 with a near Nernstian sensitivity of 55.4 mV pH^−1^ [38]. Similarly, IrO_2_ was electrodeposited onto a conductive Ag fabric as a WE to measure the open-circuit voltage variation in the WE vs. the Ag/AgCl RE due to the pH changes [95]. The fabricated sensors presented a sub-Nernstian response with a sensitivity of −47.54 mV/pH. 

In addition, the pH in sweat can affect the resistance change of conductive materials, and analyzing the change in the resistance is also a simple way to evaluate the pH in sweat. Ouyang et al. [113] coated cellulose nanocrystals (CNC)-PPy on silk–PU blended yarns with a high conductivity. As shown in Figure 5E, the resistance of the composite yarns had a full-range pH value response of at various states.

### 4.4. Glucose- and Lactate-Sensing Devices

It has been found that sweat glucose can directly reflect the blood glucose level [114]. Especially for diabetes patients who need to monitor their blood glucose level frequently and need targeted treatment, non-invasive monitoring is very important. Glucose is found in the concentration range of 0.02–0.6 mM in human sweat [72]. Monitoring lactate can prevent muscle soreness, pain and cramp during exercise [22,115]. Lactate and glucose sensors are usually enzyme-based (glucose oxidase (GOx), glucose dehydrogenase (GDH) and lactate oxidase (LOx)) sensors that respond to lactate and glucose concentrations by electrochemical measurements [35,71]. Therefore, a fabricated electrode is essential for lactate and glucose sensors, which can be integrated into yarns or fabric. Liu et al. [43] immersed carbon-coated threads in either GOx or LOx as a WE. Thread coated with Ag/AgCl ink was used for the RE. The fabricated electrodes were then embroidered on a garment to measure glucose andlactate simultaneously and produced stable electrochemical reactions. Zhao et al. [18] decorated the surface of a cotton thread with ZnO NWs as electrodes, which improved the surface of the electrodes. The strong electrostatic attraction of the ZnO NWs enabled the LOx to be fixed on their surface more effectively and stably. The lactate was oxidized by the Lox immobilized on the WE, generating and transporting free electrons between the WE and the RE, thus producing an electromotive force difference produced between the WE and the RE. Coating the electrode with Prussian blue (PB) resulted in a significant increase in glucose sensitivity, contributing to an improvement in the limit of detection, which can be explained the reduction in the overpotential required for amperometric detection by the PB mediator [116]. Elastomeric Au/SEBS fibers coated with PB/LOx and Ag were integrated into a t-shirt and served as the WE and RE, respectively, and this arrangement could maintain its high performance under a large strain of up to 100% [37]. However, the stability and long-term activation of the enzymes are the key issues that affecting the lifetime of the sensors. Liu et al. [24] processed SF into a nanofiber structure (SFNF) and cross-linked it with enzymes and glutaraldehyde to form SFNFs. Then, the SFNFs were combined with PtNPs/Gr as an electrode, which could stabilize the enzyme and facilitate electron transport between the enzymes and the electrodes. The prepared sweat sensor could achieve long-term stabilities of up to 25 h (glucose) and 23.6 h (lactate). Although enzyme-based sensors have good selectivity and high sensitivity, other factors such as the pH value, temperature, etc., will interfere with their detection and affect their accuracy [117]. 

Therefore, non-enzymatic sensors have been explored to overcome these problems during the detection of the glucose or lactate level. Several noble metals (e. g. Pt, Au) have been developed for the preparation of non-enzymatic sensors. Au structures exhibits excellent poison resistivity for the electrooxidation of glucose due to its large specific surface area and highly active binding sites on the particle surface [12,118]. Cu-based glucose sensors have attracted much attention due to their low cost and easy implementation, making them suitable as non-enzymatic alternative materials for glucose sensing [119,120]. This is due to the obvious redox reaction between Cu materials and glucose. Franco et al. [119] dipped Cu_2_O nanoclusters onto Gr paste printed on cellulose cloth as a WE. The fabricated sensors showed good sensing performance in the range from 0.1 to 1 mM glucose with a sensitivity of 182.9 ± 8.83% µA mM^−1^ cm^−2^. Metal–organic frameworks (MOFs) have been explored as non-enzymatic biosensors because of their unique catalytic activity [121]. The active sites of MOFs initiate a reaction between the molecules and produce sensing signals by converting oxidizing glucose into gluconolactone [116]. Similarly, Ppy has been coated onto the surface of MWCNTs to form core–sheath-structured NWs, which improves the electrical conductivity of MWCNTs [122]. Furthermore, Ppy is a P-type conducting polymer in which only anions are doped, resulting in a charge transfer. Accordingly, it can selectively detect lactate anions at specific potentials while being inert to neutral and cationic substances in human sweat. 

In addition to the electrochemical methods mentioned above, colorimetric methods have also been used for the preparation of glucose or lactate sensors [22,62,65]. For a glucose assay, a solution containing GOx was added to the detection area, and the color of the reference dots changed from light to dark brown with a gradient increase in concentration increasing from 10 to 2000 μM [62]. He et al. [65] distributed enzymes on paper that changed color from white to blue when glucose in sweat contacted the enzymes. The colors were then converted to R, G and B values using a smartphone for the quantitative analysis of the glucose concentration. It was found that there was a negative linear correlation between the R value and the logarithm of glucose concentration in the range from 50 to 600 μM. 

### 4.5. Other Sweat-Biomarker-Sensing Devices 

Cortisol is recognized as a stress hormone [97,123]. The concentration of cortisol in sweat changes during human daily activities [97], so it is necessary to monitor the change in cortisol concentrations in real time. The textile-based sweat sensor detection of cortisol includes enzymatic and non-enzymatic methods. Some inorganic compounds such as ZnO, TiO_2_, SnO_2_, Fe_2_O_3_, MoS_2_, etc., have been reported as enzyme-based cortisol sensors [15,36,124,125]. For example, Sekar and co-workers proposed cortisol sensors by coating Fe_2_O_3_ [124] and ZnO NRs [36] on the surface of flexible conductive carbon yarns (CCY), which exhibited a wide linear detection range and mechanical stability. In addition, ZnO NRs/CCY used as electrodes showed good biocompatibility, which could be more safely used in wearable sensors. Kinnamon et al. [15] used MoS_2_ nanosheets functionalized with cortisol antibodies as recognizable receptors. When cortisol was bound to the surface of the MoS_2_ nanosheets, the surface charge changed, and the change in the cortisol concentration could be obtained from the change in the impedance. Non-enzymatic sensors are mainly based on molecular imprinted polymers (MIPs) or recognizable receptors interacting with cortisol. As a kind of MIP, poly (glycidylmethacrylate-co ethylene glycol dimethacrylate) (poly (GMA-co-EGDMA)) can be used for the stable detection of cortisol [14,97]. Mugo et al. [14] coated AuNPs@MIP@PANI@CNT/CNC onto a cotton textile substrate via layer-by-layer assembly. CNT/CNC, PANI and AuNP layers were added to improve the conductivity, while the poly (GMA-co-EGDMA) layer ensured the selective capture of the cortisol analyte for detection. When the insulating cortisol was captured and entered the textile electrode, its capacitance was reduced. The detection limit of the prepared cortisol sensor was 8.00 ng/mL.

Urea is an important marker of the status of the human kidney. Clinical studies have shown that the uric acid concentration in sweat is highly correlated with that in the blood [126]. The direct carbonization of PAN nanofibers can produce carbon nanofibers with an oriented graphitized layer structure, which can be directly used as a working electrode for uric acid detection and contribute to the rapid transmission of electrons [126]. Different carbonation temperatures will affect the accuracy of uric acid content detection. When the carbonation temperature is 1000 ℃, the uric acid molecules in sweat can be detected sensitively. A certain concentration of urease and phenol red were successively coated on a cotton thread coated with CNF and CS-GO [23]. When the urea in sweat came into contact with the cotton thread, it changed the PH value of the cotton thread, resulting in a significant color change. 

The excessive consumption of alcohol can affect people’s spirits, cause crime in the short term and affect people’s physical health. About 1% of alcohol is metabolized by the liver, diffused by sweat through the skin and excreted by exocrine glands [127]. Some studies have demonstrated that the alcohol level in sweat can be correlated to the alcohol level in perspiration and the blood [128,129,130,131]. Some alcohol-sensing wearable sensors have been developed for detecting alcohol including enzyme (alcohol oxidase (AOx), alcohol dehydrogenase (ADH))-based electrochemical sensors [129]. Biscay et al. [129] dropped AOx onto carbon screen-printed electrodes modified with PANI, which could continuously detect alcohol in sweat in small volumes (≤40 μL). Bhide et al. [73] immobilized biotinylated AOx enzymes on a ZnO/PA membrane surface, and EIS was used to record the impedance changes without being affected by interferents. 

### 4.6. Sweat Self-Powered Batteries

**Figure 6:**
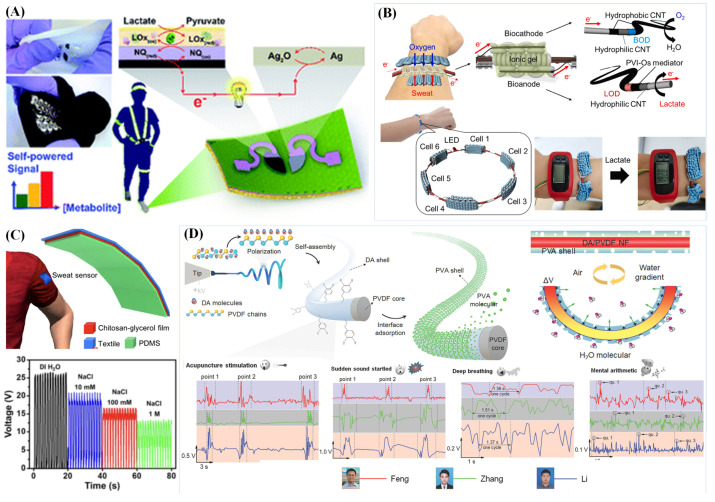
Various self-powered sweat sensors. (**A**) A stretchable BFC based on glucose or lactate with serpentine-patterned electrodes (Reprinted with permission from Ref. [84]. Copyright 2016, copyright Royal Society of Chemistry); (**B**) BFC based on lactate powered a digital wrist watch (Reprinted with permission from Ref. [132]. Copyright 2021, copyright Elsevier); (**C**) The output of liquid–solid contact TENG changed with the concentration of NaCl solution (Adapted with permission from Ref. [82]. Copyright 2018, copyright Elsevier); (**D**) A piezoelectric generator driven by a humidity actuator (Adapted with permission from Ref. [136]. Copyright 2021, copyright Small).

Wearable electronic devices generally require a power supply to work, while the development of self-powered batteries using sweat enables signal detection without an external power supply. Recently, textile-based batteries powered by sweat, including BFCs derived from the electrochemical reactions in sweat, liquid–solid contactTENG and humidity driven electric generator, have been developed. The acquisition and conversion of these energies are environmentally friendly and sustainable, and they are promising for powering miniature electronics. The metabolites in sweat, such as glucose, lactate and alcohol, react electrocatalytically with enzymes or microbes to produce energy and are used as BFCs to sense biomarkers and power electronic devices [84,85,86]. For instance, Jeerapan et al. [84] developed stretchable BFCs using electrodes with serpentine patterns printed on textiles by screen printing. As shown in Figure 6A, when the anode was functionalized with GOx or LOx and was contacted with glucose or lactate to release electrons by enzyme oxidation, the maximum open circuit voltage generated was 0.44 V, and the corresponding maximum power density was 160 μW/cm^2^. Placing six BFCs in series at the bottom of sock could directly power six LED lights. It is worth noting that the oxidation reaction at the anode is complemented by the redox reaction at the cathode [86]. BFCs based on lactate in sweat have been developed into bracelets and have been successfully used to power digital wrist watches [132]. As shown in Figure 6B, LOx/osmium-based mediator/CNF fibers were used as an anode for lactate oxidation, and bilirubin oxidase/CNF fibers were used as a cathode for oxygen reduction. Among them, the anode was optimized by an osmium-based mediator, which effectively improved the electron transfer efficiency. Six BFCs were connected using fibers to form the bracelet, generating a voltage of 2 V, which was sufficient to power a digital wristwatch. Therefore, the materials of the cathode and anode as well as the ionic conductivity between them affect the energy output of BFCs [132]. Sun et al. [84] demonstrated a BFC powered by alcohol drinkers’ perspiration. The combination of an enzymatic screen-printed electrode and a three-dimensional coralloid nitrogen-doped hierarchical micro–mesoporous carbon aerogels (NHCAs) consumed the sweat alcohol and dissolved oxygen to produce electrical bioenergy in real-time, which led to a power density of 1.9 μW cm^−2^ at 0.39 V. 

Solid–solid contact TENG systems for wearable sensors have been widely developed, which are only suitable for converting mechanical energy to electrical energy [133,134]. On other occasions, liquid–solid contact TENG systems have been established according to the change in ion concentration or sweat evaporation. Sweat-activated batteries based on the electrolytes in sweat have been developed in recent years and have the potential to be used in self-powered wearable textiles [135]. For example, Rao et al. [82] reported a flexible self-powered sweat sensor and found that a PDMS/fabric (CS-glycerol) and NaCl solution could generate surface charges during contact and separation. As depicted in Figure 6C, when the concentration of the NaCl solution changed, the amount of charge generated also changed.

The subtle change in environmental humidity caused by sweat evaporation is usually ignored by people, but it is of great value in developing a humidity-driven electric or piezoelectric generator combined with sweat humidity changes. Tong et al. [136] developed a humidity-actuator-driven piezoelectric generator (HAPG) composed of highly aligned polyvinylidene fluoride (PVDF)–PVA@dopamine (DA) core–sheath nanofibers, which showed a high sensitivity and selectivity to humidity fluctuations with excellent stability. PVA with a 50 nm thickness was coated on the surface of DA/PVDF nanofibers by vacuum filtration. When the PVA was in contact with water molecules, hydrogen bonds were spontaneously formed to cause mechanical deformation in the fibers. Therefore, mechanical energy can be effectively extracted from the fluctuation in environmental humidity and converted into electric energy, which effectively improves the sensitivity of these devices to humidity changes and avoids energy loss.

**Table 3:** Classification of sweat sensing devices.

Analyte	Sensing Element	Substrate	Preparation Method	Detection Method	Test Range	Sensitivity	Limit of Detection	Flexible	Ref.
Sweat quantity, saline concentration	-	Cotton textile as sweat collector	Screen-printed Gr-doped carbon paste electrodes	Impedance, capacitive	0–300 μL, 0–100 mM	-	-	-	[11]
Sweat quantity	Conductive threads	Cotton cover	Braiding	Conductive	0–80 mg	0.0063–0.2856 V/mg	-	0–90° bending	[19]
Lactate, Na^+^	ZnO NWs, LOx electrode, ion-selective electrode	Cotton threads	Weaving	Potentiometric	0–25 mM (lactate), 0.1–100 mM (Na^+^)	-	3.61 mM (lactate, 0.16 mM (Na^+^)	-	[18]
K^+^	PAN/PVP/Valinomycin-nylon sheath–core-structured yarns	Polyester yarns	Weaving	Potentiometric	1 × 10^−5^–2 × 10^−1^ M	34.7 mV/dec	1 × 10^−5^ M	-	[45]
Cl^−^, PH	Ag/AgCl (Cl^−^), PEDOT: BTB (pH)	PEDOT: PSS physical deposition on cotton threads	Electrodeposition	Electrochemically gated	10–150 mM (Cl^−^), 4–7 (PH)	(167 ± 3) 10^−3^ dec^−1^, (13 ± 1) 10^−3^ pH unit^−1^	-	-	[56]
pH, Cl^−^, glucose	pH indicator, HgSO_4_/FeSO_4_(Cl^−^), GOx(glucose)	Dyed cotton fabric	Embroidering	Colorimetric, RGB value	4.0–9.0, 10–150 mM (chloride), 10–2000 μM (glucose)	-	10 mM, 10 μM	-	[62]
pH, lactate	BCG and MO (PH), LOx	Knitted cotton fabrics, NaCMC, CTAB, CS	Screen-printing	Colorimetric	0–14 (PH), 0–25 mM (lactate)	-	-	-	[22]
pH	PANI	PU	Coaxial ES	Chronopotentiometry	2–7	60 mV/pH	-	122% stretching, 250° twisting or bending	[111]
pH	IrO_2_	Stainless-steel mesh	Electrodeposition	Potentiometric	4–8	−47.54 mV/pH	-	Good flexible	[95]
pH	Au, 4-MBA	PU nanofiber	ES, sputtering	Surface-enhanced Raman scattering	5.5–7.0	∼0.14–0.33 pH resolution	-	50% strain	[1]
Glucose	Cu_2_O	Cellulose paper, hand printed graphene paste electrodes	Drop casting	Voltammetric	0.1 to 1 mM	182.9 µA mM^−1^ cm^−2^	52.7	-	[119]
Glucose	Au	Carbon cloth	Electrodeposition	Amperometric	1–2164 μM	25.391 μA mM^−1^ (<114μM), 20.609 μA mM^−1^ cm^−2^ (>114 μM)	0.78 μM	-	[12]
Lactate	PPy	MWCNT	Electrodeposition	Chronoamperometric	51 μM–27.7 mM	2.9 µA mM^−1^ cm^−2^	51 µM	-	[122]
Lactate	GOx PB/Au/AuNWs-SEBS	Latex rubber core yarn covered with nylon	Electrodeposition	Chronoamperometric	0–500 μM	11.7 μA mM^−1^ cm^−2^	0	200% strain	[39]
Cortisol	ZnO nanorods	Conductive carbon yarns	Sputtering, growth	Voltammetric	1 fg/mL^–1^ μg/mL	2.12 μA/(g mL^−1^)	0.098 fg/mL	-	[36]

## 5. Conclusions and Future Scope

Massive developments in textile-based sweat sensors have shown the great potential for traditional textiles in the field of wearable electronics. As shown, textile-based sweat sensors have unique structures, a high flexibility and good air permeability, thus giving users a better wearing experience. In this short review, we introduced several types of textile-based sweat sensors for wearable applications. The main components of textile-based sweat sensors and their roles in the sensors were investigated. Several mechanisms and functions of sweat sensors in detecting biomarkers were also briefed. The flexible combination of functional materials and textiles has made great progress in the development of sweat sensors.

Human sweating depends on race, body parts, environmental temperature, physical activity and other factors. Sweat sensors are challenging for the long-term monitoring of users who have dry skin and do not sweat easily. This means that a sweat sensor with a lower detection limit is in demand. Next, the effective combination of physical and sweat sensors helps to comprehensively reflect the human body’s condition. Furthermore, the preparation of most sweat sensors is still in the laboratory testing stage, and efforts are still required to turn them into reliable commercial products. Therefore, the combination of sensing with communication and analysis technology demands more attention. The improved wireless communication range facilitates real-time and continuous signal acquisition during events such as competitions. At the same time, the integration of communication and analysis components should not affect the sensing performance and flexibility.

At present, there are several problems blocking wearable sweat sensors from being transformed into reliable commercial products. Firstly, the preparation process and material selection of the sensors lacks consideration of sustainability and environmental friendliness. In most cases, the substrate materials are synthetic materials, which can produce uncomfortable reactions and poor degradation when in long-term contact with skin. It has become a trend to develop natural materials for the preparation of wearable textile-based sweat sensors. Apart from the comfort of wearing a sweat sensor and the sensitivity of sweat detection, it is also necessary to consider whether human movement and environmental changes in the wearing process will affect the detection performance and sensitivity. Secondly, electrochemical sensors need to be calibrated repeatedly and frequently due to individual differences, making it inconvenient for public users. Next, for the preparation of non-enzymatic sensors based on electrochemical detection methods, the preparation of electrode materials with low cost, good stability and good performance is yet to be explored. Lastly, the long-term stability and durable performance of sweat sensors needs to be emphasized, since it is common that sweat sensors become unstable and inaccurate after prolonged use or being contaminated by sweat. This leads to a followed issue of the improper disposal of colorimetric sensors, showing poor environmental protection and sustainability consideration.

## Figures and Tables

**Table 1:** Comparison of different mechanisms of sweat sensors.

Methods	Mechanism	Advantages	Drawbacks	Applications	Ref.
Electrochemical detection	An analytical signal is generated through a recognition element coupled with an electrochemical transducer, providing information about the analyte concentration.	High selectivity, high accuracy and high efficiency.	Ion-selective electrodes, two or three electrodes are required, power is needed calibration is required.	Ion sensing, lactate, glucose, alcohol	[17,18]
Conductive detection	Direct measurement of the conductivity of the sensing element. The content of the substance is determined based on the signal generated by the EIS converted to a conductivity value.	No need for a reference electrode.	For EIS measurement, the information needs to be transformed according to the impedance spectrum.	Sweat quantity, ion concentration, cortisol	[11,15,19]
Colorimetric detection	The content of a substance determined by the color depth of the chromogenic reaction.	Convenient andwithout external power supply	Low accuracy, long reaction time, contrasting colors or graphics captured by users.	pH value, lactate, glucose, alcohol	[20,21,22]

**Table 2:** Common components of sweat and their associated diagnosis applications.

Components	Relative Range	Diagnosis Applications	Ref.
Average sweat rate	0.72–3.65 mg cm^−2^ min^−1^	Dehydration	[89]
Na^+^	10–100 mM	Heat stress, hyponatremia, cystic fibrosis	[17,87,90]
K^+^	1–24 mM	Hypokalemia, irregular heartbeat and arrhythmia	[45]
Cl^−^	10–100 mM	Hyper/hypo chloremia, cystic fibrosis	[62,90,91]
Ca^2+^	0.2–0.7 mM	Renal disorder, hypocalcemia	[92]
NH_4_^+^	0.1–1 mM	Metabolic breakdown of proteins	[93]
Glucose	0.02–0.6 mM	Diabetes, blood glucose	[72,94]
Lactate	5–25 mM	Muscle soreness, pain, cramp	[37]
PH	4–7	Hydration, dermatitis, ichthyosis, fungal infections	[1,95,96]
Cortisol	8–50 ng/mL	Pressure, post-traumatic-stress disorder, bipolar disorder, irritable bowel syndrome	[97]
Urea	2–10 mM	Uraemia indicating renal dysfunction	[91]
Ethanol	2.5–22.5 mM	Hypoglycemia, drinking driver	[73]

## Data Availability

Not applicable.

## References

[1] Chung M., Skinner W.H., Robert C., Campbell C.J., Rossi R.M., Koutsos V., Radacsi N. (2021). Fabrication of a Wearable Flexible Sweat pH Sensor Based on SERS-Active Au/TPU Electrospun Nanofibers. ACS Appl. Mater. Interfaces.

[2] Ghaffari R., Yang D.S., Kim J., Mansour A., Wright J.A., Model J.B., Wright D.E., Rogers J.A., Ray T.R. (2021). State of Sweat: Emerging Wearable Systems for Real-Time, Noninvasive Sweat Sensing and Analytics. ACS Sens..

[3] Baker L.B. (2019). Physiology of sweat gland function: The roles of sweating and sweat composition in human health. Temperature.

[4] Zhong B., Jiang K., Wang L., Shen G. (2022). Wearable Sweat Loss Measuring Devices: From the Role of Sweat Loss to Advanced Mechanisms and Designs. Adv. Sci..

[5] Mena-Bravo A., Luque de Castro M.D. (2014). Sweat: A sample with limited present applications and promising future in metabolomics. J. Pharm. Biomed. Anal..

[6] Wilke K., Martin A., Terstegen L., Biel S.S. (2007). A short history of sweat gland biology. Int. J. Cosmet. Sci..

[7] Sempionatto J.R., Jeerapan I., Krishnan S., Wang J. (2020). Wearable Chemical Sensors: Emerging Systems for On-Body Analytical Chemistry. Anal. Chem..

[8] Sonner Z., Wilder E., Heikenfeld J., Kasting G., Beyette F., Swaile D., Sherman F., Joyce J., Hagen J., Kelley-Loughnane N. (2015). The microfluidics of the eccrine sweat gland, including biomarker partitioning, transport, and biosensing implications. Biomicrofluidics.

[9] Gao W., Emaminejad S., Nyein H.Y.Y., Challa S., Chen K., Peck A., Fahad H.M., Ota H., Shiraki H., Kiriya D. (2016). Fully integrated wearable sensor arrays for multiplexed in situ perspiration analysis. Nature.

[10] Bariya M., Nyein H.Y.Y., Javey A. (2018). Wearable sweat sensors. Nat. Electron..

[11] Chen Y.C., Shan S.S., Liao Y.T., Liao Y.C. (2021). Bio-inspired fractal textile device for rapid sweat collection and monitoring. Lab Chip.

[12] Peng Q., Zhang Y., Yang S., Yuwen T., Liu Y., Fan J., Zang G. (2021). Glucose determination behaviour of gold microspheres-electrodeposited carbon cloth flexible electrodes in neutral media. Anal. Chim. Acta.

[13] Rachoin J.S., Weisberg L.S., McFadden C.B. (2010). Treatment of lactic acidosis: Appropriate confusion. J. Hosp. Med..

[14] Mugo S.M., Lu W., Robertson S. (2022). A Wearable, Textile-Based Polyacrylate Imprinted Electrochemical Sensor for Cortisol Detection in Sweat. Biosensors.

[15] Kinnamon D., Ghanta R., Lin K.C., Muthukumar S., Prasad S. (2017). Portable biosensor for monitoring cortisol in low-volume perspired human sweat. Sci. Rep..

[16] Promphet N., Ummartyotin S., Ngeontae W., Puthongkham P., Rodthongkum N. (2021). Non-invasive wearable chemical sensors in real-life applications. Anal. Chim. Acta.

[17] Mohan A.M.V., Rajendran V., Mishra R.K., Jayaraman M. (2020). Recent advances and perspectives in sweat based wearable electrochemical sensors. TrAC Trends Anal. Chem..

[18] Zhao C., Li X., Wu Q., Liu X. (2021). A thread-based wearable sweat nanobiosensor. Biosens. Bioelectron..

[19] Jia J., Xu C., Pan S., Xia S., Wei P., Noh H.Y., Zhang P., Jiang X. (2018). Conductive Thread-Based Textile Sensor for Continuous Perspiration Level Monitoring. Sensors.

[20] Koh A., Kang D., Xue Y., Lee S., Pielak R.M., Kim J., Hwang T., Min S., Banks A., Bastien P. (2016). A soft, wearable microfluidic device for the capture, storage, and colorimetric sensing of sweat. Sci. Transl. Med..

[21] Yin L., Cao M., Kim K.N., Lin M., Moon J.-M., Sempionatto J.R., Yu J., Liu R., Wicker C., Trifonov A. (2022). A stretchable epidermal sweat sensing platform with an integrated printed battery and electrochromic display. Nat. Electron..

[22] Promphet N., Rattanawaleedirojn P., Siralertmukul K., Soatthiyanon N., Potiyaraj P., Thanawattano C., Hinestroza J.P., Rodthongkum N. (2019). Non-invasive textile based colorimetric sensor for the simultaneous detection of sweat pH and lactate. Talanta.

[23] Promphet N., Hinestroza J.P., Rattanawaleedirojn P., Soatthiyanon N., Siralertmukul K., Potiyaraj P., Rodthongkum N. (2020). Cotton thread-based wearable sensor for non-invasive simultaneous diagnosis of diabetes and kidney failure. Sens. Actuators B Chem..

[24] Liu X., Zhang W., Lin Z., Meng Z., Shi C., Xu Z., Yang L., Liu X.Y. (2021). Coupling of Silk Fibroin Nanofibrils Enzymatic Membrane with Ultra-Thin PtNPs/Graphene Film to Acquire Long and Stable On-Skin Sweat Glucose and Lactate Sensing. Small Methods.

[25] Lu S., Wang X., Lu Q., Hu X., Uppal N., Omenetto F.G., Kaplan D.L. (2009). Stabilization of Enzymes in Silk Films. Biomacromolecules.

[26] Chen Q., Liu Y., Gu K., Yao J., Shao Z., Chen X. (2022). Silk-Based Electrochemical Sensor for the Detection of Glucose in Sweat. Biomacromolecules.

[27] Lu W., Jian M., Wang Q., Xia K., Zhang M., Wang H., He W., Lu H., Zhang Y. (2019). Hollow core-sheath nanocarbon spheres grown on carbonized silk fabrics for self-supported and nonenzymatic glucose sensing. Nanoscale.

[28] Yin J., Reddy V.S., Chinnappan A., Ramakrishna S., Xu L. (2022). Electrospun Micro/Nanofiber with Various Structures and Functions for Wearable Physical Sensors. Polym. Rev..

[29] Moharramzadeh F., Zarghami V., Mazaheri M., Simchi A. (2022). Concurrent electrophoretic deposition of enzyme-laden chitosan/graphene oxide composite films for biosensing. Mater. Lett..

[30] Lipińska W., Siuzdak K., Karczewski J., Dołęga A., Grochowska K. (2021). Electrochemical glucose sensor based on the glucose oxidase entrapped in chitosan immobilized onto laser-processed Au-Ti electrode. Sens. Actuators B Chem..

[31] Poletti F., Favaretto L., Kovtun A., Treossi E., Corticelli F., Gazzano M., Palermo V., Zanardi C., Melucci M. (2020). Electrochemical sensing of glucose by chitosan modified graphene oxide. J. Phys. Mater..

[32] Chung M., Fortunato G., Radacsi N. (2019). Wearable flexible sweat sensors for healthcare monitoring: A review. J. R. Soc. Interface.

[33] Kang X., Wang J., Wu H., Aksay I.A., Liu J., Lin Y. (2009). Glucose oxidase-graphene-chitosan modified electrode for direct electrochemistry and glucose sensing. Biosens. Bioelectron..

[34] Sun Z., Wen X., Wang L., Ji D., Qin X., Yu J., Ramakrishna S. (2022). Emerging design principles, materials, and applications for moisture-enabled electric generation. eScience.

[35] He X., Yang S., Pei Q., Song Y., Liu C., Xu T., Zhang X. (2020). Integrated Smart Janus Textile Bands for Self-Pumping Sweat Sampling and Analysis. ACS Sens..

[36] Sekar M., Allen J.A., Sriramprabha R., Pandiaraj M., Shekhar B., Ponpandian N., Viswanathan C. (2020). ZnO Nanorod Integrated Flexible Carbon Fibers for Sweat Cortisol Detection. ACS Appl. Electron. Mater..

[37] Wang R., Zhai Q., An T., Gong S., Cheng W. (2021). Stretchable gold fiber-based wearable textile electrochemical biosensor for lactate monitoring in sweat. Talanta.

[38] Napier B.S., Matzeu G., Presti M.L., Omenetto F.G. (2021). Dry Spun, Bulk-Functionalized rGO Fibers for Textile Integrated Potentiometric Sensors. Adv. Mater. Technol..

[39] Zhao Y., Zhai Q., Dong D., An T., Gong S., Shi Q., Cheng W. (2019). Highly Stretchable and Strain-Insensitive Fiber-Based Wearable Electrochemical Biosensor to Monitor Glucose in the Sweat. Anal. Chem..

[40] Zhou G., Byun J.H., Oh Y., Jung B.M., Cha H.J., Seong D.G., Um M.K., Hyun S., Chou T.W. (2017). Highly Sensitive Wearable Textile-Based Humidity Sensor Made of High-Strength, Single-Walled Carbon Nanotube/Poly(vinyl alcohol) Filaments. ACS Appl. Mater. Interfaces.

[41] Devarayan K., Kim B. (2015). Reversible and universal pH sensing cellulose nanofibers for health monitor. Sens. Actuators B Chem..

[42] Yin J., Ahmed A., Xu L. (2021). High-Throughput Free Surface Electrospinning Using Solution Reservoirs with Different Depths and Its Preparation Mechanism Study. Adv. Fiber Mater..

[43] Liu X., Lillehoj P.B. (2016). Embroidered electrochemical sensors for biomolecular detection. Lab Chip.

[44] Yin J., Xu L., Ahmed A. (2022). Batch Preparation and Characterization of Electrospun Porous Polylactic Acid-Based Nanofiber Membranes for Antibacterial Wound Dressing. Adv. Fiber Mater..

[45] Mo L., Ma X., Fan L., Xin J.H., Yu H. (2023). Weavable, large-scaled, rapid response, long-term stable electrochemical fabric sensor integrated into clothing for monitoring potassium ions in sweat. Chem. Eng. J..

[46] Stoppa M., Chiolerio A. (2016). Testing and evaluation of wearable electronic textiles and assessment thereof. Performance Testing of Textiles.

[47] Sinha A., Dhanjai, Stavrakis A.K., Stojanovic G.M. (2022). Textile-based electrochemical sensors and their applications. Talanta.

[48] Wang L., Wang L., Zhang Y., Pan J., Li S., Sun X., Zhang B., Peng H. (2018). Weaving Sensing Fibers into Electrochemical Fabric for Real-Time Health Monitoring. Adv. Funct. Mater..

[49] Stojanović G.M., Radetić M.M., Šaponjić Z.V., Radoičić M.B., Radovanović M.R., Popović Ž.V., Vukmirović S.N. (2020). A Textile-Based Microfluidic Platform for the Detection of Cytostatic Drug Concentration in Sweat Samples. Appl. Sci..

[50] Zhang J., Zhou Q., Cao J., Wu W., Zhang H., Shi Y., Mao Q., Ma H. (2021). Flexible textile ion sensors based on reduced graphene oxide/fullerene and their potential applications of sweat characterization. Cellulose.

[51] Yang S., Li C., Wen N., Xu S., Huang H., Cong T., Zhao Y., Fan Z., Liu K., Pan L. (2021). All-fabric-based multifunctional textile sensor for detection and discrimination of humidity, temperature, and strain stimuli. J. Mater. Chem. C.

[52] Parrilla M., Canovas R., Jeerapan I., Andrade F.J., Wang J. (2016). A Textile-Based Stretchable Multi-Ion Potentiometric Sensor. Adv. Healthc. Mater..

[53] Naik A.R., Zhou Y., Dey A.A., Arellano D.L.G., Okoroanyanwu U., Secor E.B., Hersam M.C., Morse J., Rothstein J.P., Carter K.R. (2021). Printed microfluidic sweat sensing platform for cortisol and glucose detection. Lab Chip.

[54] Saha S., Das S. (2021). Nanomaterials in thin-film form for new-generation energy storage device applications. Chemical Solution Synthesis for Materials Design and Thin Film Device Applications.

[55] Yang G., Kampstra K.L., Abidian M.R. (2014). High performance conducting polymer nanofiber biosensors for detection of biomolecules. Adv. Mater..

[56] Possanzini L., Decataldo F., Mariani F., Gualandi I., Tessarolo M., Scavetta E., Fraboni B. (2020). Textile sensors platform for the selective and simultaneous detection of chloride ion and pH in sweat. Sci. Rep..

[57] Choi J., Xue Y., Xia W., Ray T.R., Reeder J.T., Bandodkar A.J., Kang D., Xu S., Huang Y., Rogers J.A. (2017). Soft, skin-mounted microfluidic systems for measuring secretory fluidic pressures generated at the surface of the skin by eccrine sweat glands. Lab Chip.

[58] Son J., Bae G.Y., Lee S., Lee G., Kim S.W., Kim D., Chung S., Cho K. (2021). Cactus-Spine-Inspired Sweat-Collecting Patch for Fast and Continuous Monitoring of Sweat. Adv. Mater..

[59] Liu G., Ho C., Slappey N., Zhou Z., Snelgrove S.E., Brown M., Grabinski A., Guo X., Chen Y., Miller K. (2016). A wearable conductivity sensor for wireless real-time sweat monitoring. Sens. Actuators B Chem..

[60] Xiao G., He J., Chen X., Qiao Y., Wang F., Xia Q., Yu L., Lu Z. (2019). A wearable, cotton thread/paper-based microfluidic device coupled with smartphone for sweat glucose sensing. Cellulose.

[61] Bae C.W., Toi P.T., Kim B.Y., Lee W.I., Lee H.B., Hanif A., Lee E.H., Lee N.E. (2019). Fully Stretchable Capillary Microfluidics-Integrated Nanoporous Gold Electrochemical Sensor for Wearable Continuous Glucose Monitoring. ACS Appl. Mater. Interfaces.

[62] Zhao Z., Li Q., Chen L., Zhao Y., Gong J., Li Z., Zhang J. (2021). A thread/fabric-based band as a flexible and wearable microfluidic device for sweat sensing and monitoring. Lab Chip.

[63] Cazalé A., Sant W., Ginot F., Launay J.C., Savourey G., Revol-Cavalier F., Lagarde J.M., Heinry D., Launay J., Temple-Boyer P. (2016). Physiological stress monitoring using sodium ion potentiometric microsensors for sweat analysis. Sens. Actuators B Chem..

[64] He X., Fan C., Xu T., Zhang X. (2021). Biospired Janus Silk E-Textiles with Wet-Thermal Comfort for Highly Efficient Biofluid Monitoring. Nano Lett..

[65] He J., Xiao G., Chen X., Qiao Y., Xu D., Lu Z. (2019). A thermoresponsive microfluidic system integrating a shape memory polymer-modified textile and a paper-based colorimetric sensor for the detection of glucose in human sweat. RSC Adv..

[66] Lisak G., Arnebrant T., Ruzgas T., Bobacka J. (2015). Textile-based sampling for potentiometric determination of ions. Anal. Chim. Acta.

[67] Zhang K., Zhang J., Wang F., Kong D. (2021). Stretchable and Superwettable Colorimetric Sensing Patch for Epidermal Collection and Analysis of Sweat. ACS Sens..

[68] Wang S., Bai Y., Yang X., Liu L., Li L., Lu Q., Li T., Zhang T. (2020). Highly stretchable potentiometric ion sensor based on surface strain redistributed fiber for sweat monitoring. Talanta.

[69] Wang Z., Shin J., Park J.H., Lee H., Kim D.H., Liu H. (2020). Engineering Materials for Electrochemical Sweat Sensing. Adv. Funct. Mater..

[70] Coppedè N., Giannetto M., Villani M., Lucchini V., Battista E., Careri M., Zappettini A. (2020). Ion selective textile organic electrochemical transistor for wearable sweat monitoring. Org. Electron..

[71] He W., Wang C., Wang H., Jian M., Lu W., Liang X., Zhang X., Yang F., Zhang Y. (2019). Integrated textile sensor patch for real-time and multiplex sweat analysis. Sci. Adv..

[72] Radwan A.B., Paramparambath S., Cabibihan J.J., Al-Ali A.K., Kasak P., Shakoor R.A., Malik R.A., Mansour S.A., Sadasivuni K.K. (2021). Superior Non-Invasive Glucose Sensor Using Bimetallic CuNi Nanospecies Coated Mesoporous Carbon. Biosensors.

[73] Bhide A., Muthukumar S., Saini A., Prasad S. (2018). Simultaneous lancet-free monitoring of alcohol and glucose from low-volumes of perspired human sweat. Sci. Rep..

[74] Parrilla M., Ortiz-Gómez I., Cánovas R., Salinas-Castillo A., Cuartero M., Crespo G.A. (2019). Wearable Potentiometric Ion Patch for On-Body Electrolyte Monitoring in Sweat: Toward a Validation Strategy to Ensure Physiological Relevance. Anal. Chem..

[75] Liao J., Zhang X., Sun Z., Chen H., Fu J., Si H., Ge C., Lin S. (2022). Laser-Induced Graphene-Based Wearable Epidermal Ion-Selective Sensors for Noninvasive Multiplexed Sweat Analysis. Biosensors.

[76] Yoon J.H., Kim S.M., Eom Y., Koo J.M., Cho H.W., Lee T.J., Lee K.G., Park H.J., Kim Y.K., Yoo H.J. (2019). Extremely Fast Self-Healable Bio-Based Supramolecular Polymer for Wearable Real-Time Sweat-Monitoring Sensor. ACS Appl. Mater. Interfaces.

[77] Smith R.E., Totti S., Velliou E., Campagnolo P., Hingley-Wilson S.M., Ward N.I., Varcoe J.R., Crean C. (2019). Development of a novel highly conductive and flexible cotton yarn for wearable pH sensor technology. Sens. Actuators B Chem..

[78] Zhang T., Ran J., Ma C., Yang B. (2020). A Universal Approach to Enhance Glucose Biosensor Performance by Building Blocks of Au Nanoparticles. Adv. Mater. Interfaces.

[79] Yoon J.H., Kim S.M., Park H.J., Kim Y.K., Oh D.X., Cho H.W., Lee K.G., Hwang S.Y., Park J., Choi B.G. (2020). Highly self-healable and flexible cable-type pH sensors for real-time monitoring of human fluids. Biosens. Bioelectron..

[80] Bandodkar A.J., Gutruf P., Choi J., Lee K., Sekine Y., Reeder J.T., Jeang W.J., Aranyosi A.J., Lee S.P., Model J.B. (2019). Battery-free, skin-interfaced microfluidicelectronic systems for simultaneous electrochemical, colorimetric, and volumetric analysis of sweat. Sci. Adv..

[81] Mirza O.M., Mujlid H., Manoharan H., Selvarajan S., Srivastava G., Khan M.A. (2022). Mathematical Framework for Wearable Devices in the Internet of Things Using Deep Learning. Diagnostics.

[82] Jao Y.-T., Yang P.-K., Chiu C.-M., Lin Y.-J., Chen S.-W., Choi D., Lin Z.-H. (2018). A textile-based triboelectric nanogenerator with humidity-resistant output characteristic and its applications in self-powered healthcare sensors. Nano Energy.

[83] Zhu M., Shi Q., He T., Yi Z., Ma Y., Yang B., Chen T., Lee C. (2019). Self-Powered and Self-Functional Cotton Sock Using Piezoelectric and Triboelectric Hybrid Mechanism for Healthcare and Sports Monitoring. ACS Nano.

[84] Jeerapan I., Sempionatto J.R., Pavinatto A., You J.M., Wang J. (2016). Stretchable Biofuel Cells as Wearable Textile-based Self-Powered Sensors. J. Mater. Chem. A Mater..

[85] Sun M., Gu Y., Pei X., Wang J., Liu J., Ma C., Bai J., Zhou M. (2021). A flexible and wearable epidermal ethanol biofuel cell for on-body and real-time bioenergy harvesting from human sweat. Nano Energy.

[86] Manjakkal L., Yin L., Nathan A., Wang J., Dahiya R. (2021). Energy Autonomous Sweat-Based Wearable Systems. Adv. Mater..

[87] Ghoorchian A., Kamalabadi M., Moradi M., Madrakian T., Afkhami A., Bagheri H., Ahmadi M., Khoshsafar H. (2022). Wearable Potentiometric Sensor Based on Na0.44MnO2 for Non-invasive Monitoring of Sodium Ions in Sweat. Anal. Chem..

[88] Caldara M., Colleoni C., Guido E., Re V., Rosace G. (2016). Optical monitoring of sweat pH by a textile fabric wearable sensor based on covalently bonded litmus-3-glycidoxypropyltrimethoxysilane coating. Sens. Actuators B Chem..

[89] Taylor N.A., Machado-Moreira C.A. (2013). Regional variations in transepidermal water loss, eccrine sweat gland density, sweat secretion rates and electrolyte composition in resting and exercising humans. Extrem. Physiol. Med..

[90] Qiao Y., Qiao L., Chen Z., Liu B., Gao L., Zhang L. (2022). Wearable Sensor for Continuous Sweat Biomarker Monitoring. Chemosensors.

[91] Dang W., Manjakkal L., Navaraj W.T., Lorenzelli L., Vinciguerra V., Dahiya R. (2018). Stretchable wireless system for sweat pH monitoring. Biosens. Bioelectron..

[92] Jasiński A., Urbanowicz M., Guziński M., Bocheńska M. (2015). Potentiometric Solid-Contact Multisensor System for Simultaneous Measurement of Several Ions. Electroanalysis.

[93] Guinovart T., Bandodkar A.J., Windmiller J.R., Andrade F.J., Wang J. (2013). A potentiometric tattoo sensor for monitoring ammonium in sweat. Analyst.

[94] Olart O., Chilo J.e., Pelegri-Sebastia J.e., Barb´e K., Moer W.V. Glucose detection in human sweat using an electronic nose. Proceedings of the 35th Annual International Conference of the IEEE.

[95] Zamora M.L., Dominguez J.M., Trujillo R.M., Goy C.B., Sánchez M.A., Madrid R.E. (2018). Potentiometric textile-based pH sensor. Sens. Actuators B Chem..

[96] Legner C., Kalwa U., Patel V., Chesmore A., Pandey S. (2019). Sweat sensing in the smart wearables era: Towards integrative, multifunctional and body-compliant perspiration analysis. Sens. Actuators A Phys..

[97] Mugo S.M., Alberkant J. (2020). Flexible molecularly imprinted electrochemical sensor for cortisol monitoring in sweat. Anal. Bioanal. Chem..

[98] Yang Y., Xing S., Fang Z., Li R., Koo H., Pan T. (2017). Wearable microfluidics: Fabric-based digital droplet flowmetry for perspiration analysis. Lab Chip.

[99] Jose M., Oudebrouckx G., Bormans S., Veske P., Thoelen R., Deferme W. (2021). Monitoring Body Fluids in Textiles: Combining Impedance and Thermal Principles in a Printed, Wearable, and Washable Sensor. ACS Sens..

[100] Xu L., Zhai H., Chen X., Liu Y., Wang M., Liu Z., Umar M., Ji C., Chen Z., Jin L. (2021). Coolmax/graphene-oxide functionalized textile humidity sensor with ultrafast response for human activities monitoring. Chem. Eng. J..

[101] Salvo P., Di Francesco F., Costanzo D., Ferrari C., Trivella M.G., De Rossi D. (2010). A Wearable Sensor for Measuring Sweat Rate. IEEE Sens. J..

[102] Kil M.S., Kim S.J., Park H.J., Yoon J.H., Jeong J.M., Choi B.G. (2022). Highly Stretchable Sensor Based on Fluid Dynamics-Assisted Graphene Inks for Real-Time Monitoring of Sweat. ACS Appl. Mater. Interfaces.

[103] Glanc M., Sophocleous M., Atkinson J.K., Garcia-Breijo E. (2013). The effect on performance of fabrication parameter variations of thick-film screen printed silver/silver chloride potentiometric reference electrodes. Sens. Actuators A Phys..

[104] Patterson M.J., Galloway S.D.R., Nimmo M.A. (2000). Variations in regional sweat composition in normal human males. Exp. Physiol..

[105] Budd P. (1989). Polyelectrolytes. Comprehensive Polymer Science and Supplements.

[106] Van der Schueren L., De Clerck K. (2012). Coloration and application of pH-sensitive dyes on textile materials. Color. Technol..

[107] El-Naggar M.E., Abu Ali O.A., Saleh D.I., Abu-Saied M.A., Khattab T.A. (2021). Preparation of green and sustainable colorimetric cotton assay using natural anthocyanins for sweat sensing. Int. J. Biol. Macromol..

[108] Abdelrahman M.S., Fouda M.M.G., Ajarem J.S., Maodaa S.N., Allam A.A., Khattab T.A. (2020). Development of colorimetric cotton swab using molecular switching hydrazone probe in calcium alginate. J. Mol. Struct..

[109] Van der Schueren L., De Meyer T., Steyaert I., Ceylan O., Hemelsoet K., Van Speybroeck V., De Clerck K. (2013). Polycaprolactone and polycaprolactone/chitosan nanofibres functionalised with the pH-sensitive dye Nitrazine Yellow. Carbohydr. Polym..

[110] Yapor J.P., Alharby A., Gentry-Weeks C., Reynolds M.M., Alam A., Li Y.V. (2017). Polydiacetylene Nanofiber Composites as a Colorimetric Sensor Responding To Escherichia coli and pH. ACS Omega.

[111] Hou X., Zhou Y., Liu Y., Wang L., Wang J. (2020). Coaxial electrospun flexible PANI//PU fibers as highly sensitive pH wearable sensor. J. Mater. Sci..

[112] Yu S., Jihong M., You Y., Haobin W., Yiran Y., Haixia Z., Wei G. (2020). Wireless battery-free wearable sweat sensor powered by human motion. Sci. Adv..

[113] Ouyang Z., Xu D., Yu H.-Y., Li S., Song Y., Tam K.C. (2022). Novel ultrasonic-coating technology to design robust, highly sensitive and wearable textile sensors with conductive nanocelluloses. Chem. Eng. J..

[114] Moyer J., Wilson D., Finkelshtein I., Wong B., Potts R. (2012). Correlation between sweat glucose and blood glucose in subjects with diabetes. Diabetes Technol. Ther..

[115] Baysal G., Önder S., Göcek İ., Trabzon L., Kızıl H., Kök F.N., Kayaoğlu B.K. (2014). Microfluidic device on a nonwoven fabric: A potential biosensor for lactate detection. Text. Res. J..

[116] Yeung K.K., Huang T., Hua Y., Zhang K., Yuen M.M.F., Gao Z. (2021). Recent Advances in Electrochemical Sensors for Wearable Sweat Monitoring: A Review. IEEE Sens. J..

[117] Lee H., Hong Y.J., Baik S., Hyeon T., Kim D.H. (2018). Enzyme-Based Glucose Sensor: From Invasive to Wearable Device. Adv. Healthc. Mater..

[118] Jena B.K., Raj C.R. (2006). Enzyme-free amperometric sensing of glucose by using gold nanoparticles. Chemistry.

[119] Franco F.F., Hogg R.A., Manjakkal L. (2022). Cu_2_O-Based Electrochemical Biosensor for Non-Invasive and Portable Glucose Detection. Biosensors.

[120] Liu W., Zhao X., Dai Y., Qi Y. (2022). Study on the oriented self-assembly of cuprous oxide micro-nano cubes and its application as a non-enzymatic glucose sensor. Colloids Surf. B Biointerfaces.

[121] Wang Z., Gui M., Asif M., Yu Y., Dong S., Wang H., Wang W., Wang F., Xiao F., Liu H. (2018). A facile modular approach to the 2D oriented assembly MOF electrode for non-enzymatic sweat biosensors. Nanoscale.

[122] Choi Y.M., Lim H., Lee H.N., Park Y.M., Park J.S., Kim H.J. (2020). Selective Nonenzymatic Amperometric Detection of Lactic Acid in Human Sweat Utilizing a Multi-Walled Carbon Nanotube (MWCNT)-Polypyrrole Core-Shell Nanowire. Biosensors.

[123] Sekar M., Sriramprabha R., Sekhar P.K., Bhansali S., Ponpandian N., Pandiaraj M., Viswanathan C. (2020). Review-Towards Wearable Sensor Platforms for the Electrochemical Detection of Cortisol. J. Electrochem. Soc..

[124] Sekar M., Pandiaraj M., Bhansali S., Ponpandian N., Viswanathan C. (2019). Carbon fiber based electrochemical sensor for sweat cortisol measurement. Sci. Rep..

[125] Manickam P., Madhu S., Fernandez R.E., Viswanathan C., Bhansali S. (2017). Fabric Based Wearable Biosensor for Continuous Monitoring of Steroids. ECS Trans..

[126] Wei X., Zhu M., Li J., Liu L., Yu J., Li Z., Ding B. (2021). Wearable biosensor for sensitive detection of uric acid in artificial sweat enabled by a fiber structured sensing interface. Nano Energy.

[127] Hawthorne J.S., Wojcik M.H. (2006). Transdermal Alcohol Measurement: A Review of the Literature. Can. Soc. Forensic Sci. J..

[128] Gamella M., Campuzano S., Manso J., Gonzalez de Rivera G., Lopez-Colino F., Reviejo A.J., Pingarron J.M. (2014). A novel non-invasive electrochemical biosensing device for in situ determination of the alcohol content in blood by monitoring ethanol in sweat. Anal. Chim. Acta.

[129] Biscay J., Findlay E., Dennany L. (2021). Electrochemical monitoring of alcohol in sweat. Talanta.

[130] Sirlanci M., Rosen I.G., Wall T.L., Luczak S.E. (2019). Applying a novel population-based model approach to estimating breath alcohol concentration (BrAC) from transdermal alcohol concentration (TAC) biosensor data. Alcohol.

[131] Yang Y., Gao W. (2019). Wearable and flexible electronics for continuous molecular monitoring. Chem. Soc. Rev..

[132] Yin S., Liu X., Kaji T., Nishina Y., Miyake T. (2021). Fiber-crafted biofuel cell bracelet for wearable electronics. Biosens. Bioelectron..

[133] Niu H., Li H., Gao S., Li Y., Wei X., Chen Y., Yue W., Zhou W., Shen G. (2022). Perception-to-Cognition Tactile Sensing Based on Artificial-Intelligence-Motivated Human Full-Skin Bionic Electronic Skin. Adv. Mater..

[134] Jingcheng L., Reddy V.S., Jayathilaka W., Chinnappan A., Ramakrishna S., Ghosh R. (2021). Intelligent Polymers, Fibers and Applications. Polymers.

[135] Xiao G., Ju J., Lu H., Shi X., Wang X., Wang W., Xia Q., Zhou G., Sun W., Li C.M. (2022). A Weavable and Scalable Cotton-Yarn-Based Battery Activated by Human Sweat for Textile Electronics. Adv. Sci..

[136] Li T., Jin F., Qu M., Yang F., Zhang J., Yuan T., Dong W., Zheng J., Wang T., Feng Z.Q. (2021). Power Generation from Moisture Fluctuations Using Polyvinyl Alcohol-Wrapped Dopamine/Polyvinylidene Difluoride Nanofibers. Small.

